# Standards of evidence in chronobiology: A response

**DOI:** 10.1186/1740-3391-7-9

**Published:** 2009-07-22

**Authors:** Patrick M Fuller, Jun Lu, Clifford B Saper

**Affiliations:** 1 Department of Neurology, Program in Neuroscience, and Division of Sleep Medicine, Beth Israel Deaconess Medical Center and Harvard Medical School, Boston, USA

## Abstract

A number of recent studies have debated the existence and nature of clocks outside the suprachiasmatic nucleus that may underlie circadian rhythms in conditions of food entrainment or methamphetamine administration. These papers claim that either the canonical clock genes, or the circuitry in the dorsomedial nucleus of the hypothalamus, may not be necessary for these forms of entrainment. In this paper, we review the evidence necessary to make these claims. In particular, we point out that it is necessary to remove classical conditioning stimuli and interval timer (homeostatic) effects to insure that the remaining entrainment is due to a circadian oscillator. None of these studies appears to meet these criteria for demonstrating circadian entrainment under these conditions. Our own studies, which were discussed in detail by a recent Review in these pages by Mistlberger and colleagues, came to an opposite conclusion. However, our studies were designed to meet these criteria, and we believe that these methodological differences explain why we find that canonical clock gene *Bmal1 *and the integrity of the dorsomedial nucleus are both required to produce true circadian entrainment under conditions of restricted feeding.

## Review

The recent review by Mistlberger and colleagues [1] purports to raise a number of important questions concerning how studies in circadian biology should be performed, and what types of standards should be met. Unfortunately, rather than engaging in a debate that broadly considers issues across the field, Mistlberger and colleagues chose to focus almost entirely on criticizing our recent paper [2].

We welcome the opportunity to engage in a discussion about the methods used in circadian biology, which we believe frequently are applied in ways that confuse circadian, homeostatic, and cognitive influences. We would like to begin at that level, first by addressing a few ground rules for such debate, such as the ways in which scientists should interact, and then turn our attention to critical standards for experiments in circadian biology. Finally, we will then address the issues raised by Mistlberger et al. about our own paper, point by point, and discuss each one specifically. Our conclusion is that not only are each of these points incorrect, but that this could have been established by Mistlberger and colleagues if they had discussed these issues with us in advance.

### Part I: Overall Issues

#### 1. Scientific discourse should be collegial, open, and transparent

We believe that maintaining an open laboratory, in which colleagues are welcome to ask questions and to come visit, and to review methods and data, is critical to maintaining a scientific environment. Our laboratory, since its inception in 1981, has operated in this way. Although Dr. Fuller had some preliminary email exchanges with Dr. Mistlberger to discuss the data, not one of the eight authors of the Mistlberger review ever contacted the corresponding author on the paper (CBS) to discuss the questions that their review raises about our data or methods. We take it as axiomatic that this is necessary before making allegations about errors in data collection or presentation. As we indicate in the rest of our detailed response, we remain available to discuss these issues and demonstrate our data and methods to any scientific colleague who is interested. Scientific discourse should start there.

#### 2. No publication ever contains all of the data

This is particularly true for publications in high visibility journals, which generally require severe compression of the manuscript. If other investigators in the field would like to see additional data, these requests should go to the corresponding author. Only if the data are not forthcoming is it appropriate to cast allegations about the data collection. We will present below the information that was requested in the review by Mistlberger and colleagues. In no case does it change our results or their import.

#### 3. Critical standards for demonstration of entrainment of circadian oscillators

In our view, this is really the heart of the matter, and the reason for us to join debate in this Response.

The demonstration of entrainment of a circadian oscillator requires that a circadian pattern should persist in the absence of an external forcing stimulus. In particular, studies should be designed to avoid providing either cognitive or homeostatic forcing stimuli to animals, which could potentially produce results that appear to be circadian. These requirements have several correlates, which we describe below. We will discuss in this review nine recent papers on the role of clock genes and the dorsomedial nucleus of the hypothalamus (DMH) in entrainment to restricted feeding or methamphetamine, and the degree to which they adhere to these principles. This information is summarized in Table 1.

**Table 1:** Methods used in recent papers examining non-traditional circadian oscillators.

**Study**	**Lesion type**	**Done in DD?**	**Measure of Entrainment**	**Deprivation period**	**Homeostatic increase in measure?**
***Clock gene deletion studies in RF***					
Fuller et al., 2008 (2)	*Bmal1 *ko	DD	Tb and LMA	Yes, 24 hrs, no anticipation in RF	No, reduced Tb and LMA
Mistlberger al., 2008 (9)	*Bmal1 *ko	Mainly DD	Motion sensor	Ad lib after RF shows no entrainment	Yes, increased activity in RF
Pendergast et al., 2009 (11)	*Bmal1 *ko	LD and DD	Wheel running	Yes, 48 hrs, no clear entrainment	Yes, increased running in RF and food deprivation
Storch and Weitz, 2009 (10)	multiple clock genes	LD and DD	Wheel running	Not done	Yes, increased running in RF
					
***Clock gene deletion study in MASCO***					
Mohawk et al., 2009 (15)	Multiple clock genes	Mainly DD	Motion sensor for *Bmal1*; wheel running for rest	Not done	Yes, increased running after MA ingestion
					
***DMH lesion studies in RF***					
Gooley et al., 2006 (8)	excitotoxic	LD only	Tb and LMA	Yes, 44 hrs, after RF	No, reduced Tb and LMA in RF
Landry et al., 2006 (5)	electrolytic	LD only	Motion sensor	Yes, 51 hrs after RF	Yes, increased activity in RF
Landry et al., 2007 (4)	electrolytic	LD only	Motion sensor	Yes, 72 hrs after RF	Yes, increased activity in RF
Moriya et al., 2009 (6)	electrolytic	LD, + DD test days	Motion sensor Tb, LMA	Yes, 46 or 58 hrs, but only first day shown	Does not say (activity normalized)

A. *External cues (other than the entraining stimulus) that might provide timing stimuli to the animal should be avoided*. This might seem axiomatic. For example, the most important entraining stimulus for mammals is light. As a result, most circadian biologists would not accept any phenomenon as circadian in nature unless it was demonstrated in continuous darkness (DD).

Nevertheless, this standard is often not observed. For example in the original studies demonstrating food entrainment (see review by Stephan [3]), animals were permitted to remain on a light-dark (LD) cycle. While the use of LD insured that the light entrained rhythm and the food entrained rhythm would remain temporally separated, the light also provides a temporal cue for food presentation. A number of recent food entrainment studies including those by Mistlberger in which he has done dorsomedial hypothalamic (DMH) lesions [4,5], have continued to be performed only under LD. However, if animals are entrained under LD, and food is provided only during the light cycle (to nocturnal animals), then the animals have the opportunity to *learn cognitively *that food will appear during the light cycle. Hence, animals may show classical conditioning by increasing activities during the light cycle that are associated with feeding (see next section). This effect is clearly demonstrated in the recent paper on DMH lesions and food entrainment in mice by Moriya and colleagues [6], in which food anticipatory activity of two animals when tested in DD (their figure Eight C, activity level prior to food omission on days 7 and 14) was reduced by about 25% compared to the activity prior to feeding on the preceding days (6 and 13) when the animal was in LD (whereas the masking effects of light on activity should have caused the opposite response). In our own studies of the effects of DMH lesions on circadian rhythms, we tested rhythms of body temperature (Tb) and locomotor activity (LMA) as measured by telemetry both in *ad lib *conditions and under restricted feeding, in both LD and DD [7,8]. Similarly, the recent experiments discussed below on the effects of clock gene deletions on food entrainment [2,9-11] all include critical experiments under DD.

B. *The circadian measures that are used to demonstrate entrainment should not be ones that are directly altered by the entraining stimulus in the same way as the "entrained" responses*. For example, most circadian researchers would agree that light has masking effects on locomotor activity. Hence, no one in the field would design an experiment where the animals were exposed to a daily light cycle (e.g., in the absence of the SCN), showed masking (i.e., decreased activity during the light cycle), and claim that the SCN was not necessary for circadian rhythms of locomotor activity.

Yet this is precisely what is being done in experiments where the entraining stimulus is a restricted period of feeding opportunity (i.e., about 20 hours of starvation each day), and the output that is measured is an increase in a response that is also increased by food deprivation. This response will of course be increased toward the end of the period of starvation, regardless of any circadian entrainment. For example, the papers cited by Mistlberger et al. [1] clearly demonstrate that wheel-running and activity measured by placing an infrared motion sensor over the food bin are behaviors whose frequency is increased by food deprivation [4,5,9-11]. Thus, they tend to produce an "interval timer" effect, i.e., toward the end of a 20 hour period of food deprivation between feeding periods, when the animal is very hungry, there will be more of these behaviors, and this increase can contribute to apparent anticipatory behavior. In studies where one wants to measure the *circadian *component of food anticipation, such measures that are increased by food deprivation should be avoided.

This may seem to be a heretical position to take, given that the phenomenon of food entrainment of circadian rhythms was first described by using running-wheel activity [3,12], and that wheel-running has been widely used in studying this behavior. However, the traditional method of examining food entrainment, using a running wheel in an LD environment, includes at least three separate cues for the intact animal: (i.) a cognitive (conditioned behavior) cue to light; (ii.) a homeostatic or "interval timer" cue, which increases wheelrunning as animals become hungrier; and (iii.) a circadian cue. A great deal of effort went into establishing that food anticipatory activity as traditionally measured indeed contains a circadian component [3]. However, when one wants to *eliminate *food anticipatory responses, it is important to remove all *three *types of cues.

A number of recent studies of food entrainment have not followed this principle. Thus in the studies by Mistlberger and colleagues [4,5,9], where the measure of output was an infrared detector suspended over the food bin, or Pendergast and coworkers[11] or Storch and Weitz [10], where wheel-running activity was measured, the overall activity was *increased *in animals on restricted feeding and/or food deprivation. As a result, Pendergast et al. [11] finally concluded: "In the absence of food, heightened activity occurs regardless of the previous feeding protocol. If this is the case, we cannot rule out that *Bmal1 *is an important molecular component of the wildtype FEO, and that in the absence of *Bmal1*, the mechanism that controls the expression of FAA becomes an interval timer."

Our data support this position. We used circadian measures that are *decreased *by food deprivation (such as body temperature or general cage locomotion as measured by a telemetry transmitter [2,8]), but which under food restriction continued to find a sharp anticipatory *increase *in those measures in the hours just prior to food availability. This approach avoids the confound of an "interval timer" or homeostatic effect, and when key experiments are done in DD, isolates the circadian component of the response. Under these conditions, when the interval timer effect is removed, *Bmal1 *-/- mice have no evidence of a food anticipatory increase in Tb or general locomotor activity.

A related problem arises in a recent study on the role of clock genes in the methamphetamine-sensitive circadian oscillator (MASCO). Honma and colleagues [13] originally described the MASCO based upon putting methamphetamine (MA) into the drinking water of rats, and inducing a second free-running rhythm measured with running wheels whose period was proportional to the dose of methamphetamine, in addition to the usual 24 hour light-entrained rhythm in activity. Similar to the food entrainable oscillator, the output that was measured (running wheel activity) is increased by MA. When rats drink MA, they remain awake and active, engaging in wheel-running and increased drinking of further MA, and further wheelrunning, until the animals are exhausted and sleep (at which point they stop drinking MA for a while). This "hourglass" or interval timer effect was raised as a criticism of the MASCO phenomenon, and Honma and colleagues [14] then did the control experiment of demonstrating the MASCO after administering MA by a continuous infusion, rather than in the drinking water. This method still showed a free-running oscillator even after SCN ablation, demonstrating that MASCO entrainment indeed represents an extra-SCN clock whose function is initiated by MA. More recently, Tataroglu and colleagues [15] showed that the MASCO also shows temporal characteristics of a circadian timer. However, as with food entrainment, the presence of a circadian component to the behavior does not rule out the participation of an interval timer as well.

A recent study by Mohawk and colleagues [16] used the original method of drinking water administration of MA, and found periodic cycling of wheel-running activity, even in animals with genetic deletions of clock genes (such as *Bmal1*). Unfortunately, this study is heir to the same "hourglass" confound as the original Honma studies, and hence a critical control would be to use a continuous infusion of MA to avoid the forcing stimulus.

We have recently taken a different approach to study the MASCO. Using wildtype mice, we provide the MA daily by injection at the same time each day. This provides a precise timing stimulus for the MASCO, and permits measurement of anticipatory physiology and behavior (as with the food entrainable oscillator). Again, we use body temperature and general cage activity, as these are both at relatively low levels in the daytime, and hence a rise *in anticipation of *the MA injection represents a real circadian response, not an hourglass response.

C. *The entrained response must persist in the absence of the entraining stimulus*. The most important criterion for judging whether a response represents circadian entrainment is to eliminate the entraining stimulus for several periods at the end of the experiment and see if the response continues at the same time or phase (i.e., phase control, a prerequisite for demonstrating entrainment of an oscillator system) or, in the case of the MASCO experiment with MA in the drinking water, a persisting free-running rhythm. For the MASCO experiments above, for example, we examine the body temperature and locomotor activity for three days after the last injection of MA, and find increases that anticipate the former injection time clearly persist for at least three days. The Mohawk et al. [16] study, which claimed that MA induced circadian locomotor rhythms in mice with clock gene mutations, indicates that animals were observed after MA was stopped, but does not indicate whether the rhythms were sustained without the drug. This would have been a critical control for the claim that the MASCO is independent of known clock genes. (A "rhythm" that stopped as soon as the drug was withdrawn would not be a rhythm at all, but rather a demonstration of the "hourglass effect.")

For experiments involving food entrainment, long term deprivation at the end of the study is more difficult, as food deprivation itself can alter physiology in small rodents. At our institution, the limit permitted by the Institutional Animal Care and Use Committee for food deprivation in most rat studies is two days (e.g., Gooley et al. [8]), but for mice the limit is one day. Interestingly, none of the studies of the effects of clock gene deletions on feeding cited by Mistlberger et al[1] included a period of food deprivation immediately after restricted feeding (Table 1). Storch and Weitz [10] did not report any data beyond the period of food restriction. Mistlberger and colleagues [9] and Pendergast et al. [11] both released their animals into *ad lib *feeding for several days before a period of food deprivation. In both studies, under DD conditions, the *Bmal1 *-/- mice had no rhythm at all under either the *ad lib *or the food deprivation conditions. These experiments provide *prima facie *evidence that *Bmal1 *-/- mice do not show circadian entrainment at all, but rather show an increase in activity as they become progressively hungrier during the restricted feeding procedure (the interval timer effect).

Among studies of the effects of DMH lesions in rats on entrainment to food, all of the studies done in by Landry and colleagues [4,5], and in our own lab [8], used at least two cycles of food deprivation (Table 1). The only study done in mice, by Moriya et al. [6], indicates that a 46 or 58 hr period of food deprivation was done at the end of the study. The authors do not comment on the health of the animals, but show data only up to hour 39 in their figure, and hence do not show a second cycle of food deprivation. Interestingly, in the only DMH-lesioned mouse for which a single cycle of food deprivation was shown during DD, there apparently was no entrainment to the food (no rhythmic behavior during food omission, their figure Eight A, animal DMHX#34).

In summary, while at least 48 hours (two cycles) of food deprivation is optimal after restricted feeding to demonstrate entrainment, 24 hours of food deprivation is probably all that can be reasonably done in mice, due to their low body mass. As an alternative, Mistlberger et al. [9] and Pendergast [11] followed restricted feeding with a period of *ad lib *feeding under DD followed by a period of food deprivation. In these studies, *Bmal1 *-/- mice failed to show anticipatory behavior. We agree with Pendergast and colleagues that an "interval timer" effect could account for the rhythmic behavior during restricted feeding in these animals. We conclude that this approach may therefore provide a valid substitute for immediate food deprivation after restricted feeding.

#### 4. Proper techniques for making brain lesions and for analysis of their extent

One of the issues raised by Mistlberger and colleagues [1] is the use of lesions of the DMH in assessing its role in circadian rhythms. To understand the differences in the results of these experiments, it is necessary to consider briefly the methodology used for making and assessing the completeness of these lesions.

The use of large electrolytic lesions, which date back to the 1930's [17], disrupts fibers of passage as well as cell bodies. Because it is not possible to know where all of the axons passing through any point in the brain originate or terminate, this method by its nature induces lesions whose exact extent cannot be assessed. In addition, because the lesions destroy the brain tissue, there is always severe distortion of the remaining brain, which makes it difficult to determine what remains intact, especially around the borders of the lesion. There is a tendency to believe that "large lesions" must be effective; but such lesions may miss their intended target, and the distortion of the remaining tissue may make it impossible to determine whether the target was included in the lesion.

Cell-specific lesions were introduced in the 1970's to avoid these problems [18]. First, the lesion kills cell bodies, but not fibers of passage. Second, because the lesions cause less injury to the surrounding tissue, there is less tissue loss, and the exact borders of the lesion and the surviving cell groups within the context of the intact brain can be more clearly defined. This allows accurate quantitative assessment of which areas were damaged by the lesion, and which were not. We have used counting boxes and multivariate statistics to compare rigorously the effects of lesions with the loss of neurons in specific populations of neurons that were damaged [8,19,20]. This procedure requires large numbers of lesions, and careful analysis of each one (e.g., in the Gooley et al. study, 55 animals were used to assess the effects of lesions of the DMH vs. surrounding areas). Hence, these methods are tedious and exacting, but they also provide rigorous and unbiased procedures for assessing lesions.

In the lesion studies of the DMH cited by Mistlberger and colleagues [4-6], the lesions were done electrolytically. All three studies involved smaller numbers of animals (7 animals in [5], 6 in [4]; the actual numbers used in [6] are not clear because the numbers given in the Methods, Results, and figure legends disagree with each other, but it appears that about 15–16 animals were analyzed). The DMH lesions were judged as "complete" in the Landry studies [4] or "more than 80%" in the Moriya study [6] by attempting to determine by eye whether tissue bordering the lesions contained viable DMH neurons. More importantly, there is internal physiological evidence in all three studies that the DMH lesions were not "complete" at all.

Animals with extensive DMH cell-specific lesions [7] have a characteristic physiological signature, consisting of (i.) low levels of total daily activity (ii.) a body temperature about 0.3°C below that of normal rats; and (iii.) almost no circadian rhythm remaining in locomotor activity, wake-sleep, or feeding in a free-run in DD conditions, but (iv.) clear preservation of the circadian rhythm of Tb. The animals identified histologically as having DMH lesions in the Gooley study had these same responses [8]. In the Landry 2007 study, the animal shown in figure One E with a partial DMH ablation had levels of daily locomotor counts similar to the unlesioned animal (in their figure One A; the complete lesion animal had low activity counts, as in our studies) [4]. Review of the activity counts in their figure Two indicates that only animals DMHx1 and DMHx3 had an overall reduction in activity. Thus only two of the six animals with "complete" DMH lesions would have been considered on physiological criteria to have had a potentially complete DMH lesion. The Moriya paper found that "DMH lesioned" animals examined with motion sensors had lower daily activity counts, but only examined the circadian pattern of activity on *ad lib *feeding under LD conditions, so it is not possible to tell whether they would have met physiological criteria for a complete DMH lesion [4,6]. In the five animals examined by telemetry sensors, the animals with "DMH lesions" had a slightly higher mean Tb at all times of day (figure Nine A), which strongly suggests that the lesions by Moriya and colleagues systematically did not include the caudal dorsal part of the DMH (which contains a small cell group that is necessary to maintain normal Tb [21], and when damaged, results in a fall of baseline Tb [7,8]).

In summary, while we appreciate how difficult it is to do a lesion study of this type properly, none of the three studies by Mistlberger and colleagues [4-6] analyzed the lesion extent rigorously, either anatomically or physiologically, and there is internal evidence that many of the animals did not have adequate DMH lesions. Hence, it is not surprising that these lesions failed to eliminate food entrainment. Given the difficulty (perhaps impossibility) of doing careful histological assessment after electrolytic lesions, such animals should at least be assessed physiologically for completeness of DMH lesions before being used in studies to assess the role of the DMH in circadian rhythms.

### Part II: Specific Issues Related to the Fuller et al. Paper

The review by Mistlberger and colleagues [1] also raised a number of very specific points about the Fuller 2008 paper [2]. These require detailed responses. Our position is that none of the allegations about improper labeling or display of data are correct, and none of the issues raised would make any difference in the interpretation of our paper. In the sections below we have numbered our responses in the same order as the Mistlberger review, so that the reader can follow along and see our responses to individual points.

#### 1a. Errors in figure S3

Figure S3 was added relatively late in the review process at the request of a reviewer, and the errors in the original version escaped the notice of the authors, reviewers, and editors. They were brought to our attention by Dr. Rae Silver, who contacted the corresponding author (CBS) on July 24, 2008 to point out that the data in figure Three B were duplicated in figure S3B, but that the onset of the daily meal had been displaced. We immediately contacted Science magazine to tell them about this error, asked to withdraw this figure which used an incorrect dataset, and made a replacement figure using the correct dataset (which has been on-line since October, 2008). This also required replacing figure S3D, which was derived from the same dataset as S3B. The editors at Science subsequently pointed out that in addition a segment of data were missing from the original figure S3B. The editors of Science also contacted the Office of Scientific Integrity at Harvard Medical School, which appointed a committee, hired a consultant, and reviewed the figures and the data involved. The reason for the errors in figure S3 was that we had inadvertently used the wrong data file to make the figure. As we demonstrated to the committee, we use software that starts the recording based on computer clock time, which may not be the same as real world time (because the computers are in constant use in animal facility rooms, they are not synchronized with real world time; as a result the computer clocks either gain or lose time, and they are not adjusted for daylight savings time). So, the investigator writes down in his notebook the external world time and the computer clock time when the experiment starts, and at the end of the experiment the start time of the data file is adjusted for the actual time at which the experiment occurred. This type of file was used to make figure Three B, for example.

In addition, during the experiment the investigators download chunks of data every day or two, so that they can follow the progress of the experiment, but mainly to make sure the animals are healthy. (We record body temperature and locomotor activity, which are good indicators of overall health, so that we do not have to disturb the animals to examine them, which would also give them circadian cues.) The data are downloaded by hand, and the new data each day are appended to the existing "working file." There may be gaps in these files, if the investigator chooses a segment that does not overlap with the previous download. The gaps are filled in by "-1's", which our analysis routine plots in the actogram as a gap. The threshold temperature is the three day running mean temperature (except for the first and last two days, which are two day running means), excluding any gaps (the "-1's" are recognized by the program as a gap and not included in the mean temperature calculation). The original figures S3B and S3D were inadvertently made from the "working file" for the same animal that was used to make figure Three B. This file had not been adjusted for real world time, so that it was displaced by about 1.5 hours. It also contained a blank segment of approximately 3 hrs., which represented one of the gaps frequently found in working files. The Harvard review committee agreed that this was a human error. The revised figures were not posted online until this review was complete, and the editors at Science were informed of the results by the Harvard committee, which was the reason for the delay. We have maintained all of the files and they are available for examination by any scientist who would like to visit.

**Figure 1:**
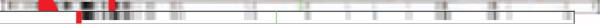
**A comparison of the data in figure 3B (upper line) and the original (incorrect) supplementary fig. S3B (lower line) in the Fuller et al. **[2]** paper, on the day in which fig. S3B contained a "gap"**. The images have been cut directly from the online figures, resized to cover the same time period, and aligned by eye. The red vertical lines marking the feeding time (the offset in the incorrect figure S3B due to not being corrected for the correct time of day) are clear. A piece of a red arrow that marks the food deprivation day is also seen toward the left in the upper register. The "gap" period is the blank area to the left of the red line in the lower register. Note that the lower register (the day in which mean body temperature was 0.06°C lower because of the missing data in the gap period) shows more time periods when the body temperature exceeded the mean (marked by gray or black boxes, depending upon how high the temperature was). Although the differences are subtle, the two plots do not "appear to be identical" as claimed by Mistlberger [1].

Mistlberger et al. [1] have further questioned why the graphs for figures Three B and S3B should "appear to be identical", if there is a segment of data missing from the datafile used to make figure S3B, claiming that the "gap" in figure S3B would cause the mean temperature for that day to be different, and hence affect the way the remaining points are plotted in the actogram. The mean temperature for the day in which the "gap" appears in the original figure S3B was 36.43°C, while the mean temperature for the same day in figure Three B, in which there is no gap was 36.49°C. Our software compares the body temperature of the animal to a running three day mean. Thus the 0.06 degree difference was averaged over three days, which were otherwise identical, and the differences in the three day rolling averages for the days that included this data in figure S3B amounted to 0.02 degrees. Another and much larger source of difference between the two graphs (figures Three B and original S3B) is that they start at different times of day, so that the actual temperature readings that constitute a "day" differ. The result is that the two graphs are not at all identical. If one compares the two at high magnification, as shown in Figure 1 in this review, there are a number of times during the day when the two differ, as would be expected for a graph produced by this thresholding method.

#### 1b. Waveforms for body temperature in figures Two and S3

The claim is made by Mistlberger et al. [1] that the fall in body temperature during the feeding period in figures Two and S3C should not occur. Our mice do not agree with this claim. In our lab, under restricted feeding conditions the intact mice (or those with *Bmal1 *gene replacement in the DMH) show a strong increase in body temperature (Tb) in anticipation of the feeding, but their Tb falls after the food is eaten, back to the levels that were sustained prior to feeding. The curves, as published, are exactly what happens. A similar fall in Tb of 1–2°C after onset of feeding has been reported by Kaur and coworkers [22] under similar conditions for C57BL6 mice in restricted feeding.

Although rats under restricted feeding in both Mistlberger and coworkers 2009 paper [23] and in our own work (Gooley et al[8], figure One D) do have increased body temperature when eating, this is not true for mice, which have a much smaller thermal mass, in a cool laboratory (22–24°C). In fact, even the mice in the Moriya study [6], in which Mistlberger was a co-author, showed a peak in Tb just before and at the time of food presentation, then a small fall, not a rise, in Tb during the remaining feeding period (e.g., see the unlesioned animal in their figure Nine A, on days 2,6, and 13 of restricted feeding; note that on days 7 and 14, when the animals were not fed, the temperature actually stayed even or rose during this period). Although the fall in Tb documented by Moriya and coworkers was smaller than in our study or that of Kaur and colleagues [22], they used a different strain of mice (ddY compared to C57BL6 in our study and that of Kaur et al.), and the thermoregulatory behavior of different mouse strains is notoriously variable.

In response to the series of questions raised by Mistlberger et al. [1] about this study: the mice were indeed fed at this time; the data are not misaligned; and they are most certainly not activity data (e.g., compare with our figure S2, which shows activity data). C57BL6 mice simply behave this way.

#### 1c. Correspondence of waveforms in figures S3C and D, with temperature "actograms" in figures S3A and B

**Figure 2:**
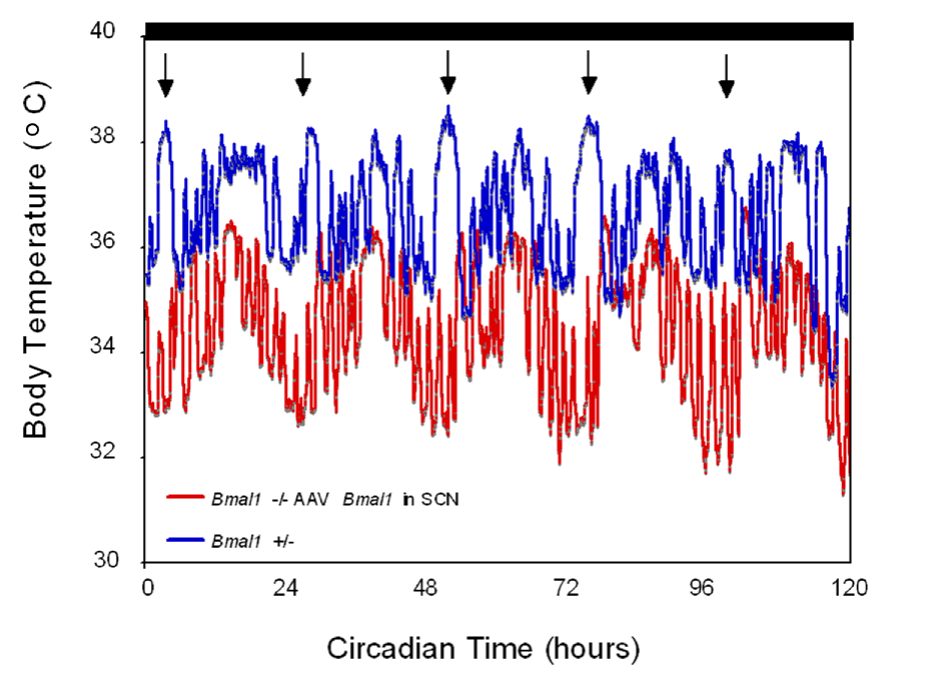
**Graphs of body temperature for the animals in the corrected suplementary figure S3 in Fuller et al**. [2]. The blue line represents the heterozygote animal shown in figures S3A and C, and the red line illustrates the *Bmal1 *-/- animal with an injection of AAV-Cre into the suprachiasmatic nucleus, shown in figures S3B and D, across the entire five day period in restricted feeding from which the summary graphs in panels C and D were derived. Note that the heterozygote animal (blue) had a normal circadian variation in body temperature, and a robust spike in temperature peaking just around the onset of time of feeding (arrows), as shown in the summary figure S3C. The animal with the injection of AAV-Cre into the suprachiasmatic nucleus had reconstitution of the daily circadian pattern, but no evidence of the anticipatory increase in body temperature prior to feeding, although there was an increase each day after feeding, consistent with the summary figure S3D.

As indicated in the response to 1a, the data in the actograms are thresholded so that temperature intervals (5 min each, so 288 per day) are indicated as dark bars when that interval is above the three day running mean (except for the first and last days, which the software program truncates to a two day running mean). The plots in panels C and D are the mean body temperature for each 5 min segment over days 10–14 of the experiment, plus or minus the SEM, which is a very different type of plot. This means that if the temperature on four days is 0.1 degrees above the mean, and on the fifth day is 1.4 degrees below the mean, the mean temperature for that time of day will be 0.2 degrees below the mean, but the actogram will show body temperature above the mean on four of five days at that time. The plots are not meant to show the data the same way, and in fact that is precisely why both types of plots were used. Both plots S3A and C were derived from the same datasets as S3B and D. We furthermore show in Figure 2 in this review the full temperature curves for these animals for all five days of recording. We would be happy to demonstrate the dataset and analysis routines to anyone who wants to try this. The claim by Mistlberger et al. that these must be misaligned or different kinds of data is simply incorrect.

#### 1d. Whether animals in figures S3A and B are in DD or LD

Mistlberger et al[1] question whether the rhythm of increased body temperature recorded during the presumptive dark cycle in these figures could have come from free-running animals. The evidence for this is supposed to be a "precise 24 hour rhythm." In fact, it is not precise at all, as even a casual inspection of the record shows, and the actual period is slightly greater than 24 hours in the animal in S3A (which is why the onset of increase is slightly later than the onset of the presumptive light cycle) and slightly less than 24 hours in the animal in S3B (which is why the onset of the increase is slightly before the presumptive light cycle, and gets earlier over the course of the experiment). Both are within the range seen for C57 mice.

### In summary

We made one unfortunate error in composing the original figure S3, which was due to inadvertently using a single incorrect data file to make the graphs S3B and D. We corrected this error as soon as possible after it was pointed out to us. All of the other issues raised by Mistlberger et al. about possible "errors in alignment or labeling of figures" are without foundation.

#### 2a. Selectivity of rescue of Bmal1 -/- mice by injection of AAV-Bmal1

**Figure 3:**
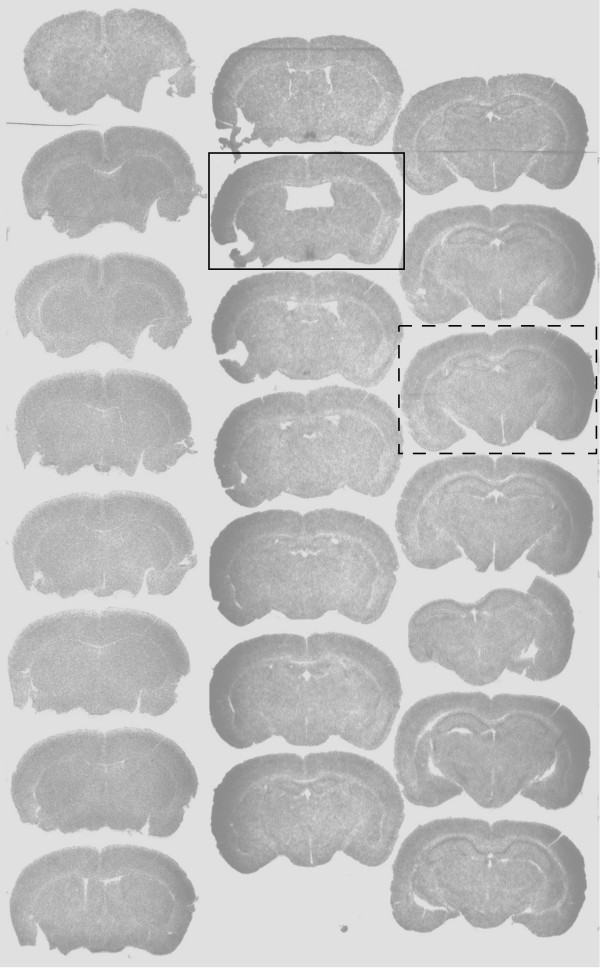
**A full set of forebrain autoradiograms on x-ray film from a *Bmal1 *-/- animal in restricted feeding who received an injection of AAV-*Bmal1 *into the suprachiasmatic nucleus bilaterally**. The box with solid lines identifies a section at the level of the SCN showing hybridization over this nucleus, and only this nucleus. The box with dashed lines represents a section at the level of the DMH, showing lack of hybridization.

**Figure 4:**
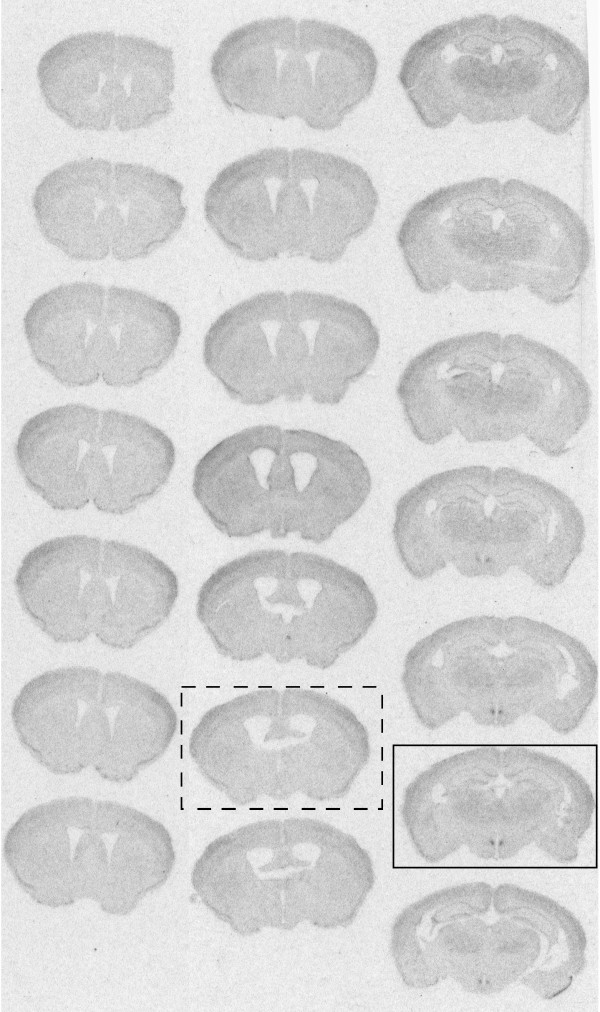
**A full set of forebrain autoradiograms on x-ray film from a *Bmal1 *-/- animal in restricted feeding with an injection of AAV-*Bmal1 *into the dorsomedial hypothalamic nucleus bilaterally**. The box with solid lines identifies a section at the level of the DMH, showing selective hybridization over this nucleus and only this nucleus. The box with dashed lines demonstrates a section at the level of the SCN, showing lack of hybridization.

Mistlberger et al. [1] raise two concerns with respect to the autoradiographs used to demonstrate that restricted feeding activates clock gene expression selectively in the DMH. The first issue is that we showed full sections for the *Per1 *hybridization, but only cropped photos of the *Bmal1 *hybridization for our rescued animals. We would point out that cropping autoradiographic images to the field of interest is quite common: Mistlberger and colleagues in the Moriya et al. [6] paper used images of autoradiograms that were cropped to show the hypothalamus in the same way as ours. The reason we did not feel it was necessary to show portions of the brain beyond the injection sites from *Bmal1 *-/- animals is that it is well known that animals without the *Bmal1 *gene do not express *Bmal1 *in the brain [24]. Showing more of the brain would only be of value to prove that the brains were not mislabeled (i.e., were not from *Bmal1 *-/- animals), as Mistlbeger et al. imply. We therefore are providing two additional figures. Figures 3 and 4 in this review show the full set of autoradiograms from the forebrains of two *Bma1I *-/- animals, one with an injection of AAV-*Bmal *into of the SCN and one into the DMH, respectively. These clearly show that the only areas of hybridization in those brains were at the injection sites.

The second concern was that the background levels of expression of *Per1 *shown in our Suplementary figure S4 in the Fuller et al. paper were similar in images shown for a *Bmal1 *+/- mouse (panel E) and a *Bmal1 *-/- mouse with a suprachiasmatic injection of AAV-*Bmal1 *(panel G). With isotopic in situ hybridization, there is always background labeling, which depends upon the exact probe used and its specific activity, stringency of washes, and sensitivity and duration of emulsion exposure. There may be differences in hybridization between different batches of probe, between slides in the same set, and even across a single slide. It is typical of autoradiograms to show higher background over areas containing large neuronal cell bodies (e.g., the pyramidal cells of the cerebral cortex or the hippocampus). This is quite apparent in the paper by Bunger et al. [24]; compare their figure Three H showing *Per2 *expression at the level of the SCN in a *Bmal1 *-/- animal, with our figure S4B in the Fuller et al. paper. Note that the Bunger paper only shows *Per1 *and *Per2 *and only at one level of the brain (the SCN). There are no figures in that paper comparable to our figures S4E or G.

In our study, the autoradiograms were done over a considerable period of time, using different batches of probe, and thus had different levels of background activity over the tissue. This study, which was started before the Mieda et al. [25] paper appeared, was initially intended to be a survey looking for cell groups with increased clock gene expression under restricted feeding, and not for quantitative mRNA measurements (see point 4b below), which is best done by Northern blots, not by autoradiography. When we found the robust activation of the DMH with food restriction, we performed semi-quantitative measurements on these images by using a ratio of the optical density of hybridization (as measured in darkfield from emulsion-dipped autoradiograms) over the DMH and SCN compared to the adjacent lateral hypothalamus, as a measure of background. (Ratios are commonly used to compare in situ hybridization autoradiograms, as they were by Mistlberger and colleagues in the Moriya et al. [6] paper, although the specific ratio procedure was not described in either paper.) However, our study was never meant to measure absolute values of clock gene expression.

The images in Supplementary figure S4 in the Fuller et al. paper and in Figures 3 and 4 in this Response, have not been adjusted for differences in background binding intensity between animals. Hence, *Per1 *in situ hybridization background over the cerebral cortex, basal ganglia, and hippocampus varies in Supplementary figure S4 panels C, E, F, and G. We cannot rule out that there may also have been some subtle variations in overall expression of *Per1 *in these brain areas these experiments, because we did not do the experiments in a way that could reliably detect those changes.

#### 2b. Were rhythms restored by injections of AAV-Bmal1 into the DMH?

Mistlberger et al. [1] raise the concern that in figure Three B in the Fuller et al. paper we do not show data on the activity patterns in a *Bmal1 *-/- animal with DMH injections of AAV-*Bmal1*, while on *ad lib *food access prior to food entrainment. This is important to establish that the animal was indeed arrhythmic prior to RF. However, the claim by Mistlberger is incorrect. The figure does in fact show data from the day before food restriction began (on the first line), and from the first day of food restriction (second line), prior to the onset of entrainment. On both days, the animal shows only the characteristic ultradian rhythms seen in completely arrhythmic *Bmal1 *knockout animals.

#### 2c. Need for 48 hour fast to demonstrate entrainment to food

This is discussed above under point 3C. While we agree that 48 hours of food deprivation would be ideal, this is probably not achievable in mice. However, we disagree with the statement that to establish that food anticipatory rhythm is "not an hourglass effect" one must remove the food for at least two cycles. If the anticipatory rhythm were an hourglass or interval timer effect, it would continue through the presumptive feeding period, as the animal became more and more hungry. If the rhythm represented circadian entrainment, it would collapse at the time of the presumptive food presentation, even though no food had been given. In our experiments (e.g., figure Three B), we found that the body temperature and activity levels after the time of presumptive food presentation was substantially lower than in the interval before it, thus supporting that this is circadian entrainment. In addition, unlike the measures that Mistlberger and colleagues have applied, the measures that we use are reduced, not increased, with starvation, and thus the elevated activity prior to food presentation cannot be due to an interval timer phenomenon.

Also, as pointed out above, Mistlberger himself has at times completely omitted food deprivation after restricted feeding (e.g., [9]) and yet claimed entrainment of *Bmal1 *-/- mice. In another paper Mistlberger co-authored with Moriya et al [6], the authors indicate that they used 46 or 58 hour food deprivation to demonstrate food entrainment of mice with DMH lesions, but show data only out to 39 hrs in their study (i.e., do *not *show the second cycle of anticipatory behavior), and the health of these animals at later time points is not indicated. It is not clear why Mistlberger considers demonstrating data from a 48 hour fast in mice to be a standard that is necessary for our work, but not his own.

#### 2d. Is there an anticipatory increase in body temperature in the Bmal1 knockout animals?

Mice show ultradian cycles in body temperature, and these are exaggerated in *Bmal1 *-/- animals. Mistlberger et al. [1] try to draw lines through these cyclic variations, which occur at random times prior to the onset of the feeding in the *Bmal1 *-/- mice (because they do not entrain). These look nothing like the robust (greater than 1.5 degrees C) increases in body temperature that are sustained over a 3 hour period prior to food presentation in wildtype mice, or those in whom *Bmal1 *has been restored in the DMH. Compare figures Two B and S3D in the Fuller et al. paper (reproduced in the Mistlberger review as their figure Eight) with figures S3A and C (Mistlberger figure Three). More importantly, the summary figure Two D in the Fuller paper [2] indicates that when the temperature is averaged for all animals over the entire three hour anticipatory time window, a method that averages out the ultradian rhythms precisely because they are *not *timed by the food presentation, the *Bmal1 *-/- animals show no anticipatory increase in temperature.

#### 2e. Did Bmal1 -/- mice lose weight in food restriction?

**Figure 5:**
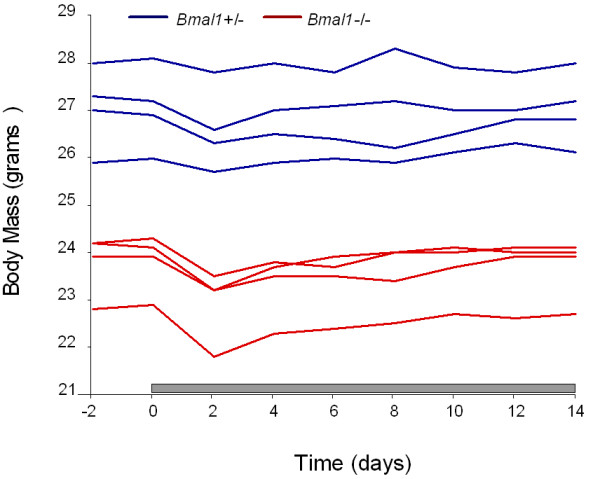
**Weights of *Bmal1 *+/- (blue) and -/- animals (red) across the food restriction experiments**. Each animal is plotted as a single line, with the heterozygotes in blue and the homozygotes in red. The *Bmal1 *-/- animals are smaller, but both groups maintain their body weight well on the restricted feeding protocol that we used.

Our animals eat about 85% of the total amount of food on food restriction as when they have food *ad lib*. This is because when the animals fail to wake up, we gently arouse them after food presentation, so that they can eat. On an *ad lib *diet the *Bmal1 *-/- mice are smaller than wildtype mice, but they *gain *weight. On food restriction they initially lose a small amount of weight (about 5%), but rapidly gain that back and at the end of the experiment are approximately the same weight as at the beginning (see Figure 5 in this review). Wildtype mice on restricted feeding also typically lose about 5% of body weight initially, gain that back, but then continue to gain weight, although at a slower rate than *ad lib *fed wildtype mice. In the two examples cited by Mistlberger et al[1] from the Yamazaki laboratory, it is not clear that the investigators actually took the precaution of awakening the mice after the onset of the food period, so they could eat. It is also noteworthy that in the experiment where the animals lost 25% of their body weight, they were only 5–8 weeks old (these are very small juvenile animals; ours were young adults, 9–11 weeks old at the start of the study). Similarly, the age of the animals in the personal communication from Nakamura is not stated. In a third experiment from Yamazaki, where the age of the mice was not reported, they lost only 8–9% of body weight. This is much closer to our experience, and indicates that the weight changes under RF vary depending upon the age of the animals, configuration of the cage, and probably many other factors (type of diet, ambient temperature, etc.). Another important factor in the reports by Yamazaki and colleagues would be whether the animals had access to running wheels as they did in the Pendergast et al. paper [11], and in other investigations such as Storch and Weitz [10]. Mohawk and colleagues[16] reported that *Bmal1 *-/- mice died (presumably from running themselves to death) when they were given access to running wheels, even when fed *ad lib*. Thus the excessive weight loss seen in labs using running wheels with *Bmal1 *-/- mice does not apply in our laboratory environment, nor does the method that we use in moving mice to restricted feeding impair their health. The observations in our lab are precisely as reported.

#### 3a. The numbers of mice used in the different experiments

in our paper [2] were as follows: Recordings of circadian behavior in *Bmal1 *-/- mice and controls: n = 12 (6 *Bmal *-/- mice and 6 *Bmal *+/- controls). Replacement of AAV-*Bmal1 *in the SCN n = 25 (6 had bilateral SCN hits; 16 missed the SCN; 3 AAV-GFP injections into the SCN were used as controls). Replacement of AAV-*Bmal1 *in the DMH n = 8 (4 had bilateral DMH hits and 4 missed the DMH). For the circadian study of clock gene expression during restricted feeding, three mice were used per time point (11 time points), per condition (*ad lib *vs. restricted feeding), or 66 mice.

#### 3b. Age of mice

As noted above, the mice used in our studies were young adults, aged 9–11 weeks at the time of surgery to implant the temperature/activity recorders. The experiments had durations of 4–6 weeks. As noted, the animals maintained their weight during the 4 hour food restriction protocol. Thus our mice were 13–17 weeks old at the end of the experiments. By contrast, the mice in the Technical Commentary on our work by Mistlberger et al. [9] were 109 ± 3 days, or 15–16 weeks *at the beginning of his experiment *which then went on for 60 days. Thus the comment that our *Bmal1-/- *animals may have failed to show entrainment because they were too old is incorrect.

#### 3c. Success rate of injection placement

Mistlberger and colleagues are correct that it is not easy to place stereotaxic injections in small hypothalamic nuclei in mice. The senior author (CBS) has been making small stereotaxic injections into the hypothalamus since 1974, however, and the technician who made the injections in this study was trained by him in 1982 and can hit the SCN bilaterally in about 25% of mice, and the DMH (which is a bigger target and easier to hit) in about 50%. The injections were fairly large, about 100 nl on each side, covering about a 800 micron sphere. As a result they either hit their target bilaterally (if the injections were at the correct AP and DV level) or missed bilaterally (if they were not). There were no unilateral hits in these series. Because *Bmal1 *was under its own promoter, it would only be expressed in cells that would normally express this gene. For example, nothing else in the SCN region normally expresses *Bmal1 *at detectable levels by autoradiography. Hence, even if the injection spills over into adjacent areas, *Bmal1 *expression is confined in the autoradiographs to the SCN. This is clear in the autoradiograms in figure One in Fuller et al. [2]. All animals with bilateral hits in SCN or DMH are reported in the Fuller et al. paper, and one of each type is shown in Figures 3 and 4 in this review. All animals with bilateral misses, as identified anatomically, had results similar to *Bmal1 *-/- animals, and were not shown in the paper.

#### 3d. Graph of Tb in groups of mice in figure Two D

This graph shows the mean temperature for each group of animals, for days 10–14 of the experiment. None of the animals showed torpor during this time window on those days when kept in DD (see next section). The full temperature curves for both of these animals for those five days are now shown in Figure 2 in this review.

#### 3e. Timing of torpor

Mistlberger et al[1] question our statement that in animals on restricted feeding during LD, more episodes of torpor occurred during the dark period. Our observations replicate the thermoregulatory behavior of mice that has been reported by multiple other laboratories. For example, Damiola et al. reported a virtually identical pattern of Tb falling into the low 30's during the dark phase when mice were fed only during the light phase [26], and Kaur et al. [22] showed a fall in Tb to about 28°C during the dark phase with restricted feeding during the light phase. Moriya et al. [6] do not show data for individual animals, but in their figure Nine illustrate a fall in mean Tb across the group down to about 33°C during the night for the group of control animals on restricted daytime feeding in LD. These are averaged data, so individual animals presumably dipped well below this temperature during periods of torpor. In the Damiola et al. experiment [26], the animals were wildtype mice that had access to food for 12 hours during the light phase, so this behavior does not represent a stress response to inadequate opportunity for feeding. In addition, the same mice did not show torpor when fed during the presumptive light phase in DD, or when fed for 12 hours a day only during the dark phase. This is simply part of the repertoire of thermal responses of mice when fed only during the day in an LD cycle in a cool laboratory. Because animals in torpor are not active, if studies of restricted feeding are done in mice on LD, without a measure of body temperature, the cycles of torpor during the dark phase under LD could be misinterpreted as circadian entrainment of activity to the light phase.

#### 3f. Ambient temperature

The rooms used for our experiments are held at 22 ± 1°C. Within these rooms, the animals are kept in isolation chambers during the experiment. We have two types of chambers in our lab: older chambers, which are larger and leak more air, in which the internal temperature is the same as the room; and newer smaller chambers with tighter fitting doors, in which the temperature during an experiment runs 24 ± 1°C. Thus both temperatures are correct in our laboratory, and the actual number depends upon which chambers were used. PMF wrote the original Supplementary Materials and gave the value for the newer chambers in which most of the studies, including all of those with AAV injections, were done. CBS revised the Supplementary Materials, and was not aware that the new chambers were different from the older ones in this regard, so changed this number back to what had previously been correct in the lab. Note that Mistlberger et al. [1] misquote us in claiming that our temperatures were ± 0.1°C. Because the range in each of the two types of boxes we use is actually ± 1°C, the two temperature ranges actually are very close and as neither temperature is anywhere near the thermoneutral temperature for mice (about 29°C), this would not have affected our results in any way.

### Conceptual issues

#### 4a. Measurement of food seeking behaviors

This is really the critical issue here. As we note above, any behavior that is increased by food deprivation should not be used for measurement of food entrainment because the results inherently are confounded by *homeostatic *responses as the animals become progressively hungrier between feedings. (This is the "interval timer" identified by Pendergast and co-workers[11].) The measures we have used, both Tb and general cage locomotion (measured with an implanted telemetry device, so that all cage movement is equally recorded) show an overall *decrease *during food deprivation both in our experiments (see Gooley et al., Table, where mean daily Tb falls from 37.50°C in ad lib to 35.07 with RF, and activity counts from 927 to 635), and in the experiments reported by Kaur et al. [22] (see their figures One and Two). Moriya et al. also found this for Tb when implanted telemetry transmitters were used [6] (their figure Nine). This is *not *the case for wheel-running [10,11], or for the infrared motion detectors used in the experiments by Mistlberger and colleagues, where food restriction routinely *increases *the levels of overall activity [4,5] (figure Three A in the Landry et al., 2007 paper shows a gradual increase in the number of counts per day for animals in RF, from about 1600 counts per day to 2000 counts per day, and this increases to 2100 counts with food deprivation).

Interestingly, in two recent papers Mistlberger and colleagues [6,23] compare the activity during restricted feeding using motion sensors as well as telemetry. In both papers, the motion counts were ''normalized (using the daily mean)'' [23], which is explained in Moyriya et al. [6] as "counts relative to the daily total, i.e., counts for each hour as a percentage of total daily counts for the day." This manipulation obscures whether the total counts are *increased *by food restriction (as they are with motion sensors), or *decreased *(as they are with telemetry). Mistlberger (2009b) claims that "overhead motion sensors and telemetry are equivalent measures of food anticipatory activity in rats." We cannot agree that these two methods measure ''the same thing'' in restricted feeding when one measure is substantially *increased *by food restriction and the other is dramatically *decreased*. Furthermore, the use of only ''normalized activity'' to hide this difference is deceptive, and should not be employed in studies comparing the two measures. Although the two measures coincide in daily *pattern *during restricted feeding in intact animals, raw counts in animals with effective DMH lesions or lack of *Bmal1 *expression would show that the two measures diverge (because the motion sensors would still detect the ''interval timer'' effect discussed by Pendergast et al. [11], while the telemetry transmitters would have no circadian signal to report). Unfortunately, the text of the report by Moriya includes only five animals with telemetry transmitters, and it is not clear from either the histology or physiology which if any of them had effective DMH lesions [6].

#### 4b. What our experiments have demonstrated

Mistlberger and colleagues consistently misrepresent what we have shown, and how we frame it. They claim that our "two studies appear to establish that the DMH contains *Bmal1*-dependent circadian oscillators that are both necessary and sufficient for the expression of food-entrainable behavioral and temperature rhythms in rodents." This is not correct. The study by Gooley et al. [8] shows that food-entrainment of wake-sleep, Tb, and LMA rhythms in rats depends upon the integrity of the DMH. We tested the role of the DMH because our previous work had shown that it was necessary to relay SCN output to control circadian rhythms of wake-sleep, locomotor activity, corticosteroid secretion, and feeding [7]. The point of the Gooley et al. paper was that the food entrainable oscillator uses the same *output *mechanisms through the DMH as does the SCN. We did not present data on the location of the food entrainable circadian oscillator in the Gooley paper, nor did we make any claims to do so.

The Fuller et al. [2] paper shows that there is activation of robust, rhythmic clock gene expression in the compact part of the DMH during restricted feeding, and this is consistent with the work of Mieda et al., [25] and Moriya et al. [6], but was the first to show that a gene from the positive limb of the clock cycle, *Bmal1*, cycles in antiphase to the *Per *genes that had been studied by Mieda [25]. Moriya et al. [6] confirm this finding. The compact part of the DMH is a separate component from the neurons involved in providing circadian output pathways (which reside in the diffuse part of the DMH [6]). The Fuller paper shows that a *Bmal1*-dependent clock gene mechanism in the DMH is *sufficient *to drive food-entrained rhythms of Tb and LMA. We specifically pointed out that there may be other clocks elsewhere in the body that are also capable of driving this rhythm. In fact, the lead sentence in the last paragraph of our paper is: "In an intact animal, peripheral oscillators in many tissues in the body, including the stomach and the liver, as well as elsewhere in the brain, may contribute to food entrainment of circadian rhythms."

Mistlberger et al. also question whether the robust, rhythmic clock gene expression in the DMH during restricted feeding is actually gene induction, rather than an increase and a shift in an existing rhythm. We agree that it is possible that there is some low level of background clock gene expression in the DMH under *ad lib *feeding. However our experiments were not designed to detect this (see section 2a above), and in fact we did not find the expression of *Per1 *or *Bmal1 *under *ad lib *feeding to differ from background in the adjacent lateral hypothalamus. The experiments of Mieda et al. [25] labeled *Per1*-positive neurons in the DMH by non-isotopic in situ hybridization. Moriya et al. misquote the Mieda paper as describing a "three-four fold higher expression near the end of the dark phase (ZT13) compared to the mid-light phase (ZT7)" for *Per1*. In fact, all of these values are cell counts, not mRNA levels, and they show fluctuations in background levels of expression, with "peaks" at ZT1 and ZT13, and troughs at ZT7 and ZT19, hardly a circadian pattern. Also because they have only two animals per time point, there is no way to identify a statistically significant rhythm from their data. Moriya et al. [6] used densitometry from x-ray film to measure clock gene expression, but provided no details on how they dealt with background or variability between animals and slides. The mRNA levels are shown as a percentage, with animals on *ad lib *feeding at ZT6 always plotted at 100%, suggesting that they were used as an internal standard for measuring a ratio. Nothing is stated about measurement of background levels of binding of the probe (e.g., in animals with clock gene knockouts). Thus, it is not clear what the low amplitude variations in levels of expression in animals that were fed *ad lib *represent. Northern blots would be preferable for measure of low levels of gene expression. As in our paper, ratios are mainly useful for providing semi-quantitative depiction of large changes in density, such as occur in the SCN and DMH, and are not suitable for measuring low level changes in gene expression (less than 2–3 fold differences).

In summary, we do not know whether there are very low levels of expression of *Per *or *Bmal1 *genes in the DMH in *ad lib *fed mice or whether they have a circadian rhythm, because the levels we observed under those conditions fell below the threshold for the method we used. Verification of a baseline level or rhythm of clock gene expression in DMH neurons in *ad lib *fed animals awaits the application of more accurate methods. Nevertheless, our observations [2] and those of Mieda et al. [25] stand that restricted feeding causes a much greater increase in expression of clock genes (*Per1, Per2*, and *Bmal1 *in appropriate phase relationship) in the DMH [2,25] and the dorsal vagal complex [25] than other sites in the brain (and no change in the SCN); that *Per *expression in the DMH begins during the time that the animals become active in anticipation of feeding (with *Bmal1 *expression in antiphase to this) [2,25]; and that the clock genes in the DMH continue to cycle for several days after they are activated, even in the absence of feeding (whereas those in dorsal vagal complex do not [25]). Finally, no other brain area shows anywhere near this robust level of clock gene activation, a finding that Moriya et al. [6] also supported.

Does this mean that no other clocks in the brain or body participate in food entrainment of circadian rhythms? No, we have never claimed that nor would we, because it has not been tested yet.

## Conclusion

We are deeply disappointed that the "review" by Mistlberger et al. [1] purports to address "Standards of evidence in chronobiology," but barely touches the surface of this important problem. Instead, the authors use the opportunity to attack our work and raise a series of baseless (and needless) accusations (which we address in the last part of our Response).

We have used the first part of our Response to return to the original problem, to identify a set of "standards of evidence in chronobiology," and have reviewed the work cited by Mistlberger and colleagues as disagreeing with us, in light of these standards. When viewed in this way, we believe that the results across the field are explainable by differences in methodology. In particular, use of activity measures that are increased during food deprivation results in preservation of food anticipatory activity in animals with DMH lesions or clock gene mutations, because of an "interval timer" effect, rather than persistence of a circadian oscillator. In addition, lesion studies require careful and rigorous controls and lesion characterization, which can only be applied when cell-specific lesions are used. When that standard is applied, the studies cited by Mistlberger et al. as showing food entrainment in DMH lesioned rats and mice are not valid, because the animals never had adequate documentation of DMH lesions in the first place.

We stand by our findings that the DMH is necessary for organizing food entrained circadian rhythms, and that under restricted feeding there is robust activation of high levels of rhythmic expression of clock genes in the DMH, which is sufficient to restore food entrained rhythms. We do not know whether there are other clocks elsewhere in the body that are capable of driving the DMH output neurons (as they are usually driven by the SCN clock during *ad lib *feeding) and shaping circadian rhythms during food entrainment. There remains a great deal to be learned about the organization of circadian rhythms by the brain, and we hope that this will be done in the spirit of collegial and open exchange of information, and with the same high standards for evidence applied to *all *of the work in the field.

## Competing interests

The authors declare that they have no competing interests.

## Authors' contributions

All authors contributed to the writing of this article. All authors read and approved the final manuscript.

## References

[B1] Mistlberger RE, Buijs RM, Challet E, Escobar C, Landry GJ, Kalsbeek A (2009). Standards of evidence in chronobiology: critical review of a report that restoration of Bmal1 expression in the dorsomedial hypothalamus is sufficient to restore circadian food anticipatory rhythms in Bmal1-/- mice. J Circadian Rhythms.

[B2] Fuller PM, Lu J, Saper CB (2008). Differential rescue of light- and food-entrainable circadian rhythms. Science.

[B3] Stephan FK (2002). The "other" circadian system: food as a Zeitgeber. J Biol Rhythms.

[B4] Landry GJ, Yamakawa GR, Webb IC, Mear RJ, Mistlberger RE (2007). The dorsomedial hypothalamic nucleus is not necessary for the expression of circadian food-anticipatory activity in rats. J Biol Rhythms.

[B5] Landry GJ, Simon MM, Webb IC, Mistlberger RE (2006). Persistence of a behavioral food-anticipatory circadian rhythm following dorsomedial hypothalamic ablation in rats. Am J Physiol Regul Integr Comp Physiol.

[B6] Moriya T, Aida R, Kudo T, Akiyama M, Doi M, Hayasaka N (2009). The dorsomedial hypothalamic nucleus is not necessary for food-anticipatory circadian rhythms of behavior, temperature, or clock gene expression in mice. Eur J Neurosci.

[B7] Chou TC, Scammell TE, Gooley JJ, Gaus SE, Saper CB, Lu J (2003). Critical role of dorsomedial hypothalamic nucleus in a wide range of behavioral circadian rhythms. J Neurosci.

[B8] Gooley JJ, Schomer A, Saper CB (2006). The dorsomedial hypothalamic nucleus is critical for the expression of food-entrainable circadian rhythms. Nat Neurosci.

[B9] Mistlberger RE, Yamazaki S, Pendergast JS, Landry GJ, Takumi T, Nakamura W (2008). Comment on "Differential rescue of light- and food-entrainable circadian rhythms". Science.

[B10] Storch KF, Weitz CJ (2009). Daily rhythms of food-anticipatory behavioral activity do not require the known circadian clock. Proc Natl Acad Sci USA.

[B11] Pendergast JS, Nakamura W, Friday RC, Hatanaka F, Takumi T, Yamazaki S (2009). Robust food anticipatory activity in BMAL1-deficient mice. PLoS ONE.

[B12] Richter CP (1922). A behavioristic study of the activity of the rat. Comp Psychol Monogr.

[B13] Honma K, Honma S (1986). Effects of methamphetamine on development of circadian rhythms in rats. Brain Dev.

[B14] Honma K, Honma S, Hiroshige T (1987). Activity rhythms in the circadian domain appear in suprachiasmatic nuclei lesioned rats given methamphetamine. Physiol Behav.

[B15] Tataroglu O, Davidson AJ, Benvenuto LJ, Menaker M (2006). The methamphetamine-sensitive circadian oscillator (MASCO) in mice. J Biol Rhythms.

[B16] Mohawk JA, Baer ML, Menaker M (2009). The methamphetamine-sensitive circadian oscillator does not employ canonical clock genes. Proc Natl Acad Sci USA.

[B17] Ranson SW (1939). Somnolence caused by hypothalamic lesions in monkeys. Arch Neurol Psychiatr.

[B18] Olney JW, Price MT (1983). Excitotoxic amino acids as neuroendocrine research tools. Methods Enzymol.

[B19] Lu J, Greco MA, Shiromani P, Saper CB (2000). Effect of lesions of the ventrolateral preoptic nucleus on NREM and REM sleep. J Neurosci.

[B20] Lu J, Zhang YH, Chou TC, Gaus SE, Elmquist JK, Shiromani P (2001). Contrasting effects of ibotenate lesions of the paraventricular nucleus and subparaventricular zone on sleep-wake cycle and temperature regulation. J Neurosci.

[B21] Nakamura K, Morrison SF (2007). Central efferent pathways mediating skin cooling-evoked sympathetic thermogenesis in brown adipose tissue. Am J Physiol Regul Integr Comp Physiol.

[B22] Kaur S, Thankachan S, Begum S, Blanco-Centurion C, Sakurai T, Yanagisawa M (2008). Entrainment of temperature and activity rhythms to restricted feeding in orexin knock out mice. Brain Res.

[B23] Mistlberger RE, Kent BA, Landry GJ (2009). Phenotyping food entrainment: motion sensors and telemetry are equivalent. J Biol Rhythms.

[B24] Bunger MK, Wilsbacher LD, Moran SM, Clendenin C, Radcliffe LA, Hogenesch JB (2000). Mop3 is an essential component of the master circadian pacemaker in mammals. Cell.

[B25] Mieda M, Williams SC, Richardson JA, Tanaka K, Yanagisawa M (2006). The dorsomedial hypothalamic nucleus as a putative food-entrainable circadian pacemaker. Proc Natl Acad Sci USA.

[B26] Damiola F, Le MN, Preitner N, Kornmann B, Fleury-Olela F, Schibler U (2000). Restricted feeding uncouples circadian oscillators in peripheral tissues from the central pacemaker in the suprachiasmatic nucleus. Genes Dev.

